# “Exported” Deaths and Short-Term PM_10_ Exposure: Factoring the Impact of Commuting into Mortality Estimates

**DOI:** 10.1289/ehp.123-A22

**Published:** 2015-01-01

**Authors:** Julia R. Barrett

**Affiliations:** Julia R. Barrett, MS, ELS, is a Madison, WI–based science writer and editor. She is a member of the National Association of Science Writers and the Board of Editors in the Life Sciences.

Exposure to coarse particulate matter (PM_10_) has been associated with increased mortality.^1^^,^^2^^,^^3^ Reliable health impact assessments are difficult, however, because existing exposure data may be incomplete, and exposures and effects alike typically are predicted rather than observed.^4^^,^^5^ A new report in *EHP* estimates mortality attributable to short-term PM_10_ exposure using sophisticated models to account for two of the chief obstacles to assessing health impact—namely, data uncertainty and mobility of the population.^4^

The study area in Lombardy is characterized by thermal inversions that trap air pollution at ground level within the highly populated Po River basin. Levels of PM_10_ in the basin often exceed guidelines set by the World Health Organization (WHO) and European Union (EU)—annual means of 20 μg/m^3^ and 40 μg/m^3^, respectively.^4^^,^^6^ For example, average annual concentrations in 2003–2006 reached 52.5 μg/m^3^ in the regional capital of Milan and 45.4 μg/m^3^ in other highly populated areas.^6^

**Figure:**
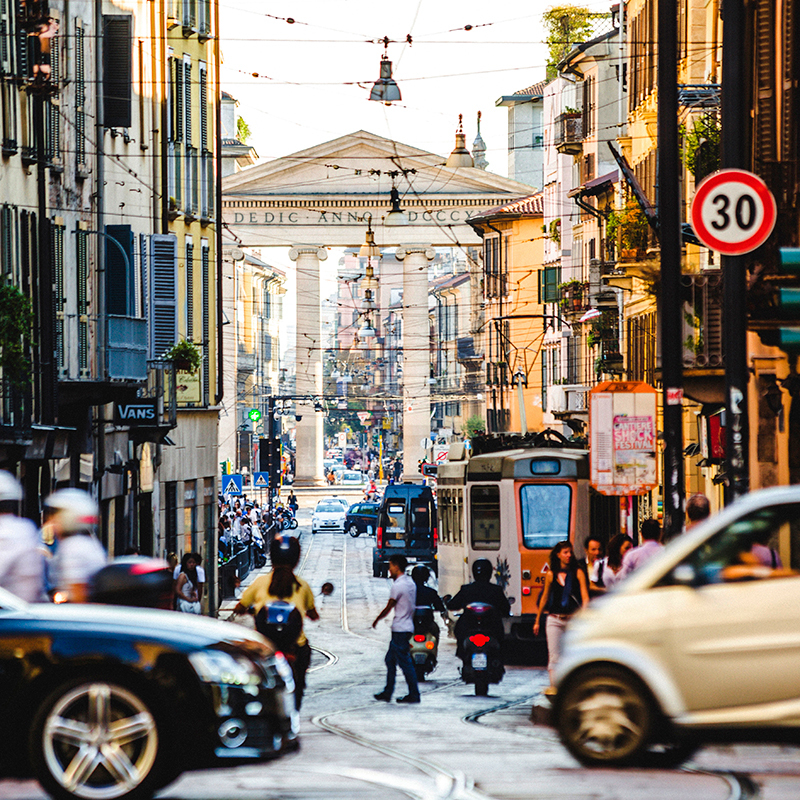
Evening rush hour in Milan, Italy. © Peeter Viisimaa/iStockphoto.com

PM_10_ is a complex mix of small particles and adsorbed substances emitted by vehicles, industrial activities, and other sources.^1^ Inhalation of PM_10_ can trigger oxidative stress, inflammation, and other physiologic reactions,^1^ and both short- and long-term exposure have been associated with cardiac and respiratory morbidity and mortality.^2^ Although PM_10_ exposure plays a relatively small role in these conditions, many people are exposed, so the public health burden builds up.^1^^,^^4^

Individual monitoring is cost prohibitive, so PM_10_ exposure is typically estimated using data from monitoring stations, modeling, and satellite images.^4^ However, uncertainty surrounding the validity or meaning of these data can undermine the reliability of the resulting health impact assessments.^5^ In addition, exposure assessments typically have not accounted for PM_10_ exposure in multiple places. For instance, although some assessments are based on residential address, people who commute to work or school may spend a large part of their day in an area more polluted than their home neighborhood.^7^

To overcome these hurdles, the authors of the current study constructed models using existing data on total mortality, PM_10_ concentrations, PM_10_ health effects, and commuting patterns among towns in the Lombardy region of Italy. Uncertainty was incorporated for parameters including variability in exposure risk between larger municipalities and smaller, less well-characterized locations. The researchers applied statistical procedures, including Bayesian techniques and Monte Carlo simulations, to address the uncertainty and pull the data into sharper focus.

The researchers estimated that in 2007, 865 deaths in Lombardy were attributable to PM_10_ concentrations exceeding the WHO standard of 20 μg/m^3^, and 26% of those deaths were attributable to PM_10_ levels above the EU standard of 40 μg/m^3^. They further estimated that annual average PM_10_ levels of 20 μg/m^3^ or lower would have resulted in 311.4 fewer deaths, while annual average PM_10_ levels of 40 μg/m^3^ or lower would have prevented 189.4 deaths.^4^

The researchers partitioned the estimated deaths based on where exposure was predicted to have occurred.^4^ “We found the health impact of air pollution is not uniform in the region but is concentrated in the capital city and other major cities,” says coauthor Michela Baccini, an associate professor in the Department of Statistics, Informatics, and Applications “G. Parenti” at the University of Florence. “Moreover, we found that air pollution in the largest cities also has an impact on the health of commuters from other municipalities in the region.” In other words, people who lived in less-polluted areas could die of exposures received in more-polluted areas, which the authors referred to as “exported” deaths.

Potential weaknesses include the fact that people who are capable of commuting may be younger and healthier than average, so the authors’ use of mortality rates and effect estimates based on the general population may have inflated the apparent impact of commuting. They also did not consider the impact of commuting within cities but assumed all exposures within a municipality were the same.

The large credibility intervals reflect the level of uncertainty factored into the model. Nevertheless, the overall picture remains intact, even though the finer details may remain murky.

“It’s a very interesting paper and solid statistical work,” says Evangelia Samoli, an assistant professor in the Department of Hygiene, Epidemiology and Medical Statistics at the University of Athens Medical School, who was not involved with the study. “I believe the main advantage of the method is the health impact assessment at the municipality level as compared to previous approaches.” This kind of small-area estimation may not be useful for informing policies on a large scale, but it does highlight the magnitude and complexity of the problem, she says.

“Our research points out that in an interconnected world it is difficult to be immune from the negative effect of pollution,” says Baccini. “Even if our residence place is ‘clean,’ commuting to work and study places can expose us to air pollution. This highlights the need to develop adequate mobility planning, but also to better plan our lifestyle and the way we live in our cities.”
